# Long-Term Outcomes With Nivolumab Plus Ipilimumab or Nivolumab Alone Versus Ipilimumab in Patients With Advanced Melanoma

**DOI:** 10.1200/JCO.21.02229

**Published:** 2021-11-24

**Authors:** Jedd D. Wolchok, Vanna Chiarion-Sileni, Rene Gonzalez, Jean-Jacques Grob, Piotr Rutkowski, Christopher D. Lao, C. Lance Cowey, Dirk Schadendorf, John Wagstaff, Reinhard Dummer, Pier Francesco Ferrucci, Michael Smylie, Marcus O. Butler, Andrew Hill, Ivan Márquez-Rodas, John B. A. G. Haanen, Massimo Guidoboni, Michele Maio, Patrick Schöffski, Matteo S. Carlino, Céleste Lebbé, Grant McArthur, Paolo A. Ascierto, Gregory A. Daniels, Georgina V. Long, Tuba Bas, Corey Ritchings, James Larkin, F. Stephen Hodi

**Affiliations:** ^1^ Memorial Sloan Kettering Cancer Center and Weill Cornell Medical College, New York, NY; ^2^ Venteo Institute of Oncology IOV–IRCCS, Padua, Italy; ^3^ University of Colorado Cancer Center, Aurora, CO; ^4^ Aix-Marseille University, APHM Timone France, Marseille, France; ^5^ Maria Sklodowska-Curie National Institute of Oncology Center, Warsaw, Poland; ^6^ University of Michigan, Ann Arbor, MI; ^7^ Texas Oncology-Baylor Charles A. Sammons Cancer Center, Dallas, TX; ^8^ University of Essen, Essen, and German Cancer Consortium, Heidelberg, Germany; ^9^ The College of Medicine, Swansea University, Swansea, United Kingdom; ^10^ Universitäts Spital Zürich, Zürich, Switzerland; ^11^ European Institute of Oncology IRCCS, Milan, Italy; ^12^ Cross Cancer Institute, Edmonton, Alberta, Canada; ^13^ Princess Margaret Cancer Centre, Toronto, Ontario, Canada; ^14^ Tasman Oncology Research, Southport, Queensland, Australia; ^15^ Hospital General Universitario Gregorio Marañon, Madrid, Spain; ^16^ Netherlands Cancer Institute, Amsterdam, the Netherlands; ^17^ Istituto Scientifico Romagnolo per lo Studio e la Cura dei Tumori IRCCS, Meldola, Italy; ^18^ Center for Immuno-Oncology, University Hospital of Siena, Siena, Italy; ^19^ University Hospitals Leuven, Department of General Medical Oncology, Leuven Cancer Institute, KU Leuven, Leuven, Belgium; ^20^ Westmead and Blacktown Hospitals, University of Sydney, Melanoma Institute Australia, Sydney, Australia; ^21^ Université de Paris, Department of Dermatology and CIC, AP-HP Hôpital Saint Louis, INSERM U976, Paris, France; ^22^ Peter MacCallum Cancer Centre, East Melbourne, Australia; ^23^ Melanoma Cancer Immunotherapy and Innovative Therapy Unit, Istituto Nazionale Tumori IRCCS Fondazione Pascale, Napoli, Italy; ^24^ UC San Diego Health – La Jolla, La Jolla, CA; ^25^ Melanoma Institute Australia, The University of Sydney, and Royal North Shore and Mater Hospitals, Sydney, Australia; ^26^ Bristol Myers Squibb, Princeton, NJ; ^27^ The Royal Marsden Hospital NHS Foundation Trust, London, United Kingdom; ^28^ Dana-Farber Cancer Institute, Boston, MA

## Abstract

**PURPOSE:**

In the phase III CheckMate 067 trial, durable clinical benefit was demonstrated previously with nivolumab plus ipilimumab and nivolumab alone versus ipilimumab. Here, we report 6.5-year efficacy and safety outcomes.

**PATIENTS AND METHODS:**

Patients with previously untreated unresectable stage III or stage IV melanoma were randomly assigned 1:1:1 to receive nivolumab 1 mg/kg plus ipilimumab 3 mg/kg once every 3 weeks (four doses) followed by nivolumab 3 mg/kg once every 2 weeks (n = 314), nivolumab 3 mg/kg once every 2 weeks (n = 316), or ipilimumab 3 mg/kg once every 3 weeks (four doses; n = 315). Coprimary end points were progression-free survival and overall survival (OS) with nivolumab plus ipilimumab or nivolumab versus ipilimumab. Secondary end points included objective response rate, descriptive efficacy assessments of nivolumab plus ipilimumab versus nivolumab alone, and safety. Melanoma-specific survival (MSS; descriptive analysis), which excludes deaths unrelated to melanoma, was also evaluated.

**RESULTS:**

Median OS (minimum follow-up, 6.5 years) was 72.1, 36.9, and 19.9 months in the combination, nivolumab, and ipilimumab groups, respectively. Median MSS was not reached, 58.7, and 21.9 months, respectively; 6.5-year OS rates were 57%, 43%, and 25% in patients with *BRAF*-mutant tumors and 46%, 42%, and 22% in those with *BRAF*–wild-type tumors, respectively. In patients who discontinued treatment, the median treatment-free interval was 27.6, 2.3, and 1.9 months, respectively. Since the 5-year analysis, no new safety signals were observed.

**CONCLUSION:**

These 6.5-year CheckMate 067 results, which include the longest median OS in a phase III melanoma trial reported to date and the first report of MSS, showed durable, improved clinical outcomes with nivolumab plus ipilimumab or nivolumab versus ipilimumab in patients with advanced melanoma and, in descriptive analyses, with the combination over nivolumab monotherapy.

## INTRODUCTION

The development of immune checkpoint inhibitors and BRAF- and MEK-targeted therapies has led to a transformation of survival outcomes in patients with advanced melanoma.^1^ In particular, the programmed cell death 1 inhibitors nivolumab and pembrolizumab and the cytotoxic T-lymphocyte–associated protein-4 blocking antibody ipilimumab have reshaped immunologic approaches to the treatment of this disease. Evidence from the randomized controlled, double-blind phase II CheckMate 069 and phase III CheckMate 067 trials established the combination of nivolumab and ipilimumab as a standard care option for patients with metastatic melanoma.^2-7^

**CONTEXT**

**Key Objective**
Long-term survival of patients with advanced melanoma treated with nivolumab plus ipilimumab and nivolumab alone versus ipilimumab has been demonstrated after 5-year follow-up in the phase III CheckMate 067 trial. The extent of the durability of this benefit is of interest. We present 6.5-year results from CheckMate 067, including a first report of melanoma-specific survival.
**Knowledge Generated**
Prolonged survival can be attained in patients with melanoma treated with nivolumab plus ipilimumab or nivolumab alone with median overall survival of 72.1 and 36.9 months, and median melanoma-specific survival not reached and 58.7 months, respectively. The majority of these patients are off treatment and have not yet started systemic subsequent therapy.
**Relevance**
These results represent a substantial development in the melanoma treatment landscape versus the standard median survival of 8 months a decade ago. This advance is supported by the ability to distinguish melanoma-specific from overall survival.

A 5-year follow-up analysis of the CheckMate 067 trial demonstrated durable clinical benefit with nivolumab plus ipilimumab or nivolumab alone compared with ipilimumab monotherapy.^7^ At 5 years, median overall survival (OS) was not reached in patients in the combination therapy group and was 36.9 and 19.9 months in the nivolumab and ipilimumab monotherapy groups, respectively. Five-year OS rates were 52%, 44%, and 26% in the three groups, respectively, and among patients with *BRAF*-mutant advanced melanoma, 5-year OS rates were 60%, 46%, and 30%, respectively. Noting that the study was not designed to compare the nivolumab-containing treatment groups, these results also suggested improved clinical outcomes with combination therapy over nivolumab monotherapy. Compared with either monotherapy group, treatment with the combination also resulted in higher proportions of patients who were alive and treatment-free at 5 years. Little is known about the long-term outcomes of nivolumab plus ipilimumab or nivolumab alone in melanoma beyond 5 years. In the current report, we describe an analysis of efficacy and safety in CheckMate 067 at 6.5 years of follow-up, which is the longest follow-up of a phase III trial with an anti-programmed cell death 1–based treatment in melanoma. In addition to reporting updates on long-term survival and safety, our analysis included melanoma-specific survival (MSS), which is increasingly valuable in removing the non–disease-related deaths that become an increasingly important consideration with long-term follow-up.

## PATIENTS AND METHODS

### Patients

Eligible adult patients had previously untreated and unresectable or metastatic histologically confirmed stage III or stage IV melanoma with known *BRAF* V600 mutation status and an Eastern Cooperative Oncology Group performance status of 0 or 1. Trial eligibility criteria have been described in detail previously.^4-6^ All patients provided written informed consent.

### Study Design, Treatment, and Assessments

The design of this trial and the assessments used have been detailed previously.^4-6^ In brief, patients were randomly assigned (1:1:1) to receive nivolumab 1 mg/kg plus ipilimumab 3 mg/kg once every 3 weeks for four doses, followed by nivolumab 3 mg/kg once every 2 weeks; nivolumab 3 mg/kg once every 2 weeks; or ipilimumab 3 mg/kg once every 3 weeks for four doses. All treatment regimens were appropriately placebo-matched for the purposes of blinding. Stratification factors were *BRAF* mutation status, American Joint Committee on Cancer (seventh edition) metastasis stage, and tumor programmed cell death ligand 1 status. Treatment was discontinued in the event of progressive disease (PD), the occurrence of unacceptable toxic events, or withdrawal of consent. Treatment could be continued beyond PD on the basis of clinical benefit without substantial adverse events (AEs) per investigator decision.

Progression-free survival (PFS; investigator-assessed) and OS in the combination or the nivolumab group compared with the ipilimumab group were coprimary end points. Secondary end points included comparison of investigator-assessed objective response rates (ORRs) in the nivolumab-containing groups and the ipilimumab group, comparison of efficacy in the combination and the nivolumab groups (descriptive analyses), and safety. MSS (which excludes deaths unrelated to melanoma) and survival outcomes by best overall response were descriptive post hoc analyses. AEs were defined using the Medical Dictionary for Regulatory Activities version 23.0 and graded on the basis of Common Terminology Criteria for Adverse Events version 4.0. Immune-mediated AEs, defined as events for which immune-modulating medication was initiated (except for endocrine events, which did not require immune-modulating medication initiation to be included), and select AEs (defined as events with a potential immunologic cause in the categories of skin, gastrointestinal, hepatic, pulmonary, renal, hypersensitivity or infusion reaction, or endocrine) were evaluated. Additional exploratory analyses (survival outcomes in other subgroups, treatment-free interval [TFI; the time from the last dose of study drug to subsequent systemic therapy initiation or the last known date alive, excluding patients who had discontinued study follow-up or had died before receiving subsequent systemic therapy], and treatment-free status) have been described previously.^5,6^

The trial was conducted in accordance with the Declaration of Helsinki and with Good Clinical Practice as defined by the International Conference on Harmonisation. The study was conducted in compliance with the Protocol (online only), which was approved by each study center's institutional review board.

### Statistical Analysis

Efficacy end points were analyzed in the intent-to-treat population, and formal analysis of the coprimary end points was conducted as reported previously.^4,5^ Updated *P* values accompanying the current 6.5-year follow-up assessments of PFS, OS, and ORR were descriptive in nature. Updated rates accompanying assessments of PFS and OS were included where the numbers of patients at risk allowed. The study was not designed or powered for a formal comparison between the nivolumab plus ipilimumab and the nivolumab treatment groups. In a post hoc analysis of MSS, events were defined as deaths because of melanoma; deaths because of other causes were censored. Additional details about the statistical analyses have been published previously.^4-6^

## RESULTS

### Patients

**FIG 1.**
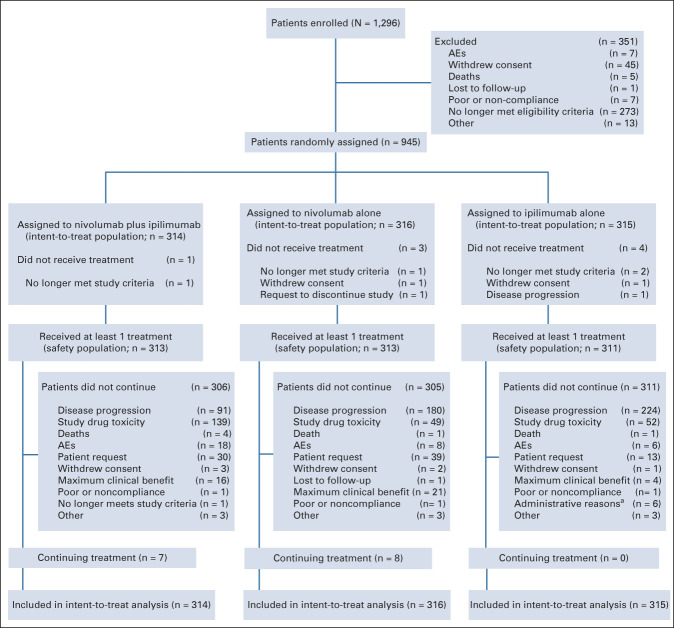
CONSORT diagram. AE, adverse event. ^a^Since the 3-year analysis, patients were unmasked and seven patients discontinued from maintenance nivolumab placebo (six for “administrative reasons” and one for “other reasons”; this latter patient is included within the three patients in this group with a reason for treatment discontinuation as “other”).

Of the 1,296 patients enrolled between July 2013 and March 2014, 945 were randomly assigned (314 to combination therapy, 316 to nivolumab, and 315 to ipilimumab; Fig 1). Patient characteristics were well balanced (Data Supplement, online only), as previously reported.^4-7^ At the time of the data cutoff on October 19, 2020, minimum follow-up for the study was 77 months from the date of the first dose of the last patient to be randomly assigned, and median follow-up (defined as the median time between the first dose and date of death or last known alive for each patient) was 57.5, 36.0, and 18.6 months in the combination, nivolumab, and ipilimumab groups, respectively. At the time of the data cutoff, most patients were off therapy (15 patients were continuing treatment: seven in the combination group and eight in the nivolumab group; Fig 1).

### Efficacy

**FIG 2.**
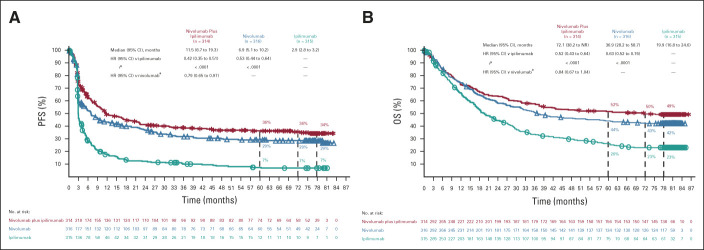
(A) PFS and (B) OS in patients who received nivolumab plus ipilimumab, nivolumab, or ipilimumab. Patients were followed for a minimum of 77 months. All rates are based on the current 6.5-year analysis; rates shown at earlier time points may differ slightly from those of previous reports. ^a^Descriptive analysis. HR, hazard ratio; NR, not reached; OS, overall survival; PFS, progression-free survival.

**FIG 3.**
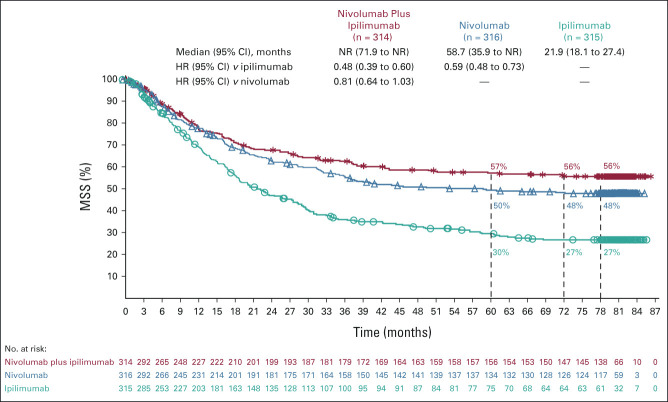
MSS in patients who received nivolumab plus ipilimumab, nivolumab, or ipilimumab. In this descriptive post hoc analysis, an event was defined as death as a result of melanoma; deaths as a result of any other causes were censored. HR, hazard ratio; MSS, melanoma-specific survival; NR, not reached.

At 77 months' minimum follow-up, both PFS and OS were longer in the nivolumab-containing treatment groups than in the ipilimumab group. Median investigator-assessed PFS (95% CI) was 11.5 months (8.7 to 19.3) in the combination group, 6.9 months (5.1 to 10.2) in the nivolumab group, and 2.9 months (2.8 to 3.2) in the ipilimumab group, with 6.5-year PFS rates of 34%, 29%, and 7%, respectively (Fig 2A). Median OS (95% CI) was 72.1 months (38.2 to not reached [NR]) in the combination group, 36.9 months (28.2 to 58.7) in the nivolumab group, and 19.9 months (16.8 to 24.6) in the ipilimumab group, with 6.5-year OS rates of 49%, 42%, and 23%, respectively (Fig 2B). In a descriptive post hoc analysis, median MSS (defined as death caused by melanoma, with deaths resulting from other causes censored) was NR, 58.7, and 21.9 months in the three groups, respectively; 6.5-year MSS rates were 56%, 48%, and 27%, respectively (Fig 3). The number and causes of nonmelanoma deaths are shown in the Data Supplement.

Between the previous 5-year analysis^7^ and the current data cutoff, a total of 11 patients died, all for reasons considered to be unrelated to treatment (Data Supplement). Of these 11 patients, three, three, and two died as a result of melanoma (with PD having been detected before the 5-year analysis) in the combination, nivolumab, and ipilimumab groups, respectively. In addition, in the nivolumab group, one patient each died from adenocarcinoma and gallbladder malignancy, and in the ipilimumab group, the cause of one patient's death was unknown.

**FIG 4.**
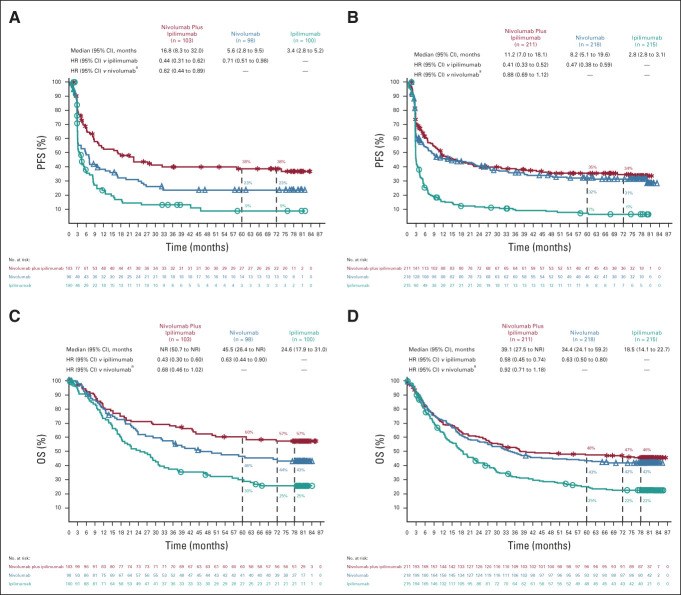
PFS in patients with (A) *BRAF*-mutant or (B) *BRAF*–wild-type tumors and OS in patients with (C) *BRAF*-mutant or (D) *BRAF*–wild-type tumors. Patients were followed for a minimum of 77 months. In the nivolumab plus ipilimumab, nivolumab, and ipilimumab groups, 314, 316, and 315 patients had *BRAF* mutational status results, respectively. ^a^Descriptive analysis. HR, hazard ratio; NR, not reached; OS, overall survival; PFS, progression-free survival.

**FIG 5.**
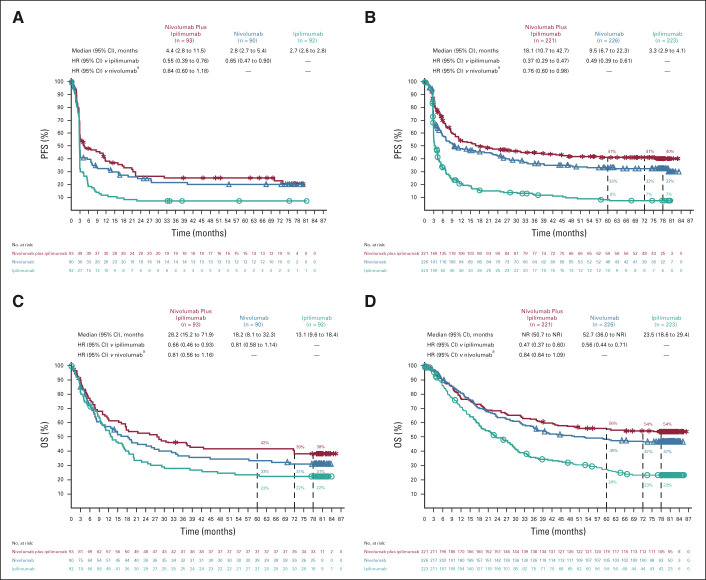
PFS in patients (A) with or (B) without baseline liver metastases and OS in patients (C) with or (D) without baseline liver metastases. Patients were followed for a minimum of 77 months. ^a^Descriptive analysis. HR, hazard ratio; NR, not reached; OS, overall survival; PFS, progression-free survival.

Long-term PFS and OS were also evaluated in a variety of clinically relevant patient subgroups. In patients with *BRAF*-mutant tumors, 6-year PFS rates (low numbers of patients at risk at 6.5 years precluded reporting 6.5-year values) were 38%, 23%, and 9% with the combination, nivolumab, and ipilimumab, respectively (Fig 4A); rates were 34%, 31%, and 6% in patients with *BRAF*–wild-type tumors, respectively (Fig 4B). OS rates at 6.5 years were 57%, 43%, and 25% in patients with *BRAF*-mutant tumors (Fig 4C) and 46%, 42%, and 22% in patients with *BRAF*–wild-type tumors, respectively (Fig 4D). Median OS was NR, 45.5, and 24.6 months in patients with *BRAF*-mutant tumors and 39.1, 34.4, and 18.5 months in patients with *BRAF*–wild-type tumors, respectively. Survival outcomes in patients with or without liver metastases at baseline are shown in Figure 5. Although numbers of patients at risk in these subgroups were small in some of the treatment groups, PFS and OS trends suggested clinical benefit in the nivolumab-containing treatment groups, particularly, the combination-therapy group, compared with the ipilimumab group regardless of baseline liver metastases. OS results at 6.5 years in patient subgroups on the basis of tumor programmed cell death ligand 1 expression level were consistent with those reported previously (Data Supplement).^5-7^

**FIG 6.**
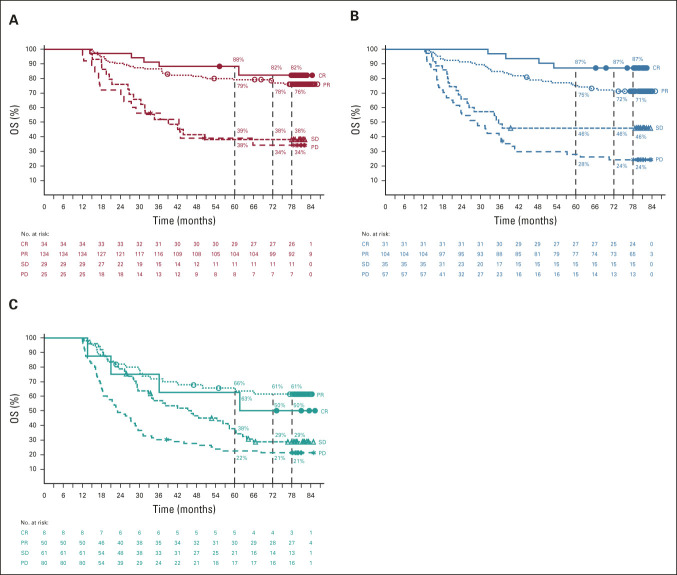
OS by best overall response in patients in the (A) nivolumab plus ipilimumab, (B) nivolumab, or (C) ipilimumab groups. Patients with a best overall response of CR, PR, SD, or PD within 12 months of treatment initiation were followed for OS. To address guarantee-time bias, these landmark analyses excluded patients who had OS events (deaths) during the first 12 months; thus, OS remained at 100% for the first 12 months in this subpopulation. CR, complete response; OS, overall survival; PD, progressive disease; PR, partial response; SD, stable disease.

ORRs at 6.5 years remained unchanged from the previous analysis at 5 years,^7^ with rates of 58%, 45%, and 19% with the combination, nivolumab, and ipilimumab, respectively (Data Supplement). Median duration of response had not been reached at 77 months in both nivolumab-containing treatment groups and was 19.2 months in the ipilimumab group. Among patients with PD, the central nervous system was the site of initial progression in 14 of 155 patients (9%) in the combination group, 19 of 184 patients (10%) in the nivolumab group, and 28 of 235 patients (12%) in the ipilimumab group (Data Supplement). Between the 5- and 6.5-year data cutoff, seven patients experienced PD (combination, n = 3; nivolumab, n = 3; and ipilimumab, n = 1). A post hoc 12-month landmark analysis (to reduce guarantee-time bias^8^) evaluated PFS and OS by best overall response (Data Supplement, Fig 6). In both nivolumab-containing groups, high rates of PFS and OS were observed in patients who had an objective response and were progression-free or alive at 12 months, respectively.

**FIG 7.**
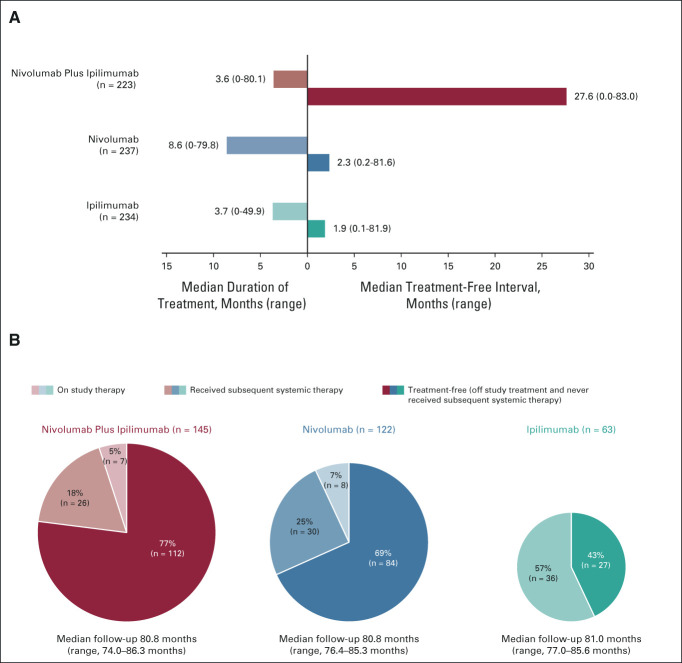
Treatment-free interval and treatment status in patients alive at 6.5 years. (A) Median treatment-free interval in each treatment group following discontinuation of study treatment. Among 313 patients treated with nivolumab plus ipilimumab, 223 were included in the analysis and 90 were excluded (seven were still on study treatment; 54 had died, six were no longer in follow-up, and six withdrew consent, all without having received subsequent therapy; 17 were excluded for other reasons). Among 313 patients treated with nivolumab, 237 were included and 76 were excluded (eight were still on study treatment; 45 had died, two were no longer in follow-up, and three withdrew consent, all without having received subsequent therapy; 18 were excluded for other reasons). Among 311 patients treated with ipilimumab, 234 patients were included and 77 were excluded (57 had died, two were no longer in follow-up, and seven withdrew consent, all without having received subsequent therapy; 11 were excluded for other reasons). Median duration of treatment in these groups is also shown. (B) Treatment status in patients alive at 6.5 years. In the combination group, 145 patients were alive and still in follow-up at 6.5 years and were included in this analysis, and 168 patients were excluded (138 had died, six were lost to follow-up, seven withdrew consent, and 17 were excluded for other reasons). In the nivolumab group, 122 patients were included and 191 patients were excluded (164 had died, six were lost to follow-up, three withdrew consent, and 18 were excluded for other reasons). In the ipilimumab group, 63 patients were included and 248 patients were excluded (218 had died, six were lost to follow-up, 13 withdrew consent, and 11 were excluded for other reasons).

As reported previously,^7^ a smaller proportion of patients in the combination therapy group received subsequent systemic therapy than did those in the nivolumab or ipilimumab groups (36%, 49%, and 66%, respectively; Data Supplement). Median (95% CI) time from random assignment to start of subsequent systemic therapy was NR (59.6 to NR), 25.2 months (16.0 to 43.2), and 8.0 months (95% CI, 6.5 to 8.7) in the three groups, respectively. The median TFI (the time from the last dose of study drug to initiation of subsequent systemic therapy or the last known date alive) was 27.6, 2.3, and 1.9 months in the three groups, respectively (Fig 7A). Among patients who were alive at the data cutoff, 77%, 69%, and 43% in the three groups were treatment-free, respectively (off study treatment, without having received any subsequent systemic therapy; Fig 7B).

### Safety

As expected, no new safety signals were detected. Updated treatment-related adverse events (TRAEs), immune-mediated AEs, and select TRAE data, including time to onset and resolution, are presented in the Data Supplement.

## DISCUSSION

These 6.5-year data with the combination of first-line nivolumab plus ipilimumab in the pivotal CheckMate 067 study include the longest median OS (72.1 months) reported to date in a phase III study of patients with advanced melanoma. For the first time, we are also able to report MSS in this population (median not reached at 77 months and 6.5-year rate of 56% with the combination), which is important, given the increasing competing risk of death from other causes that the durable control of melanoma with checkpoint inhibitors affords. At 6.5 years, median duration of response had yet to be reached with both nivolumab-containing regimens, and only three patients in either group had experienced PD since the 5-year analysis. Durable clinical benefit was observed across clinically relevant subgroups, including those based on baseline *BRAF* mutation or baseline liver metastasis status. Less than half of the patients treated with the combination received any subsequent therapy, and the median TFI was 27.6 months with the combination (*v* 2.3 months with nivolumab and 1.9 months with ipilimumab). Of patients alive at 6.5 years, 77% treated with the combination and 69% treated with nivolumab were treatment-free. No new safety signals were observed, no treatment-related deaths had occurred since the 28-month analysis, and only 11 deaths of any cause had occurred across the three treatment groups since the 5-year analysis.

Long-term survival analysis in this study continued to show substantially improved OS with nivolumab alone or in combination with ipilimumab compared with ipilimumab alone. Although the study was not powered to compare nivolumab plus ipilimumab and nivolumab alone, median OS with the combination was approximately twice as long as with nivolumab alone, supporting a meaningful survival benefit of the combination compared with nivolumab monotherapy. The durability of the long-term survival benefit was demonstrated by the continued plateaus of the survival curves with longer follow-up. Although subsequent therapies may have contributed, in part, to these plateaus, it is noteworthy that the majority of patients who were alive at 6.5 years were treatment-free in the nivolumab-containing treatment groups. The 72-month median OS and 49% 6.5-year OS rate achieved with nivolumab plus ipilimumab represent an impressive development in the melanoma treatment landscape when compared with the median OS of 8 months and 10% 5-year OS rate that was the standard 10 years ago.^9^ The fact that it is now possible to discriminate between MSS (median not yet reached with nivolumab plus ipilimumab combination therapy) and OS results further attests to this development. Seven patients across all three treatment groups experienced PD between the 5- and 6.5-year analyses. Of 11 deaths since the 5-year analysis overall, eight resulted from PD and three from other causes unrelated to treatment. Noting the problems inherent in cross-trial comparisons, the current results compare favorably with those obtained with other standard first-line treatments for advanced melanoma, such as pembrolizumab, for which a 5-year OS rate of 43.2% has been reported as first-line treatment.^10^ Five-year OS rates of 31% to 35% have been reported with the combination of BRAF and MEK inhibitors in *BRAF*-mutant melanoma.^11-13^

With long-term follow-up, the nivolumab-containing regimens in this study continued to demonstrate survival benefit over ipilimumab alone irrespective of *BRAF* mutational status. Long-term follow-up also confirmed the trend of continued separation between the combination and nivolumab monotherapy curves in patients with *BRAF*-mutant disease that has been observed previously,^6,7^ noting that the study was not designed to formally compare these treatment groups or subgroups. The 6.5-year OS rate of 57% and median OS that had not been reached in patients with *BRAF-*mutant disease in the nivolumab plus ipilimumab group demonstrates the efficacy of this treatment in this patient population and highlights the important question of what represents optimal first-line therapy and treatment sequencing for these patients, a question that awaits OS results of trials such as SECOMBIT (NCT02631447) and DREAMseq (NCT02224781) for more definitive resolution.

In the current analysis, nivolumab-containing regimens, particularly the combination, also demonstrated long-term clinical benefit versus ipilimumab in patient subgroups on the basis of the presence or absence of liver metastases at baseline. Survival outcomes overall were poorer in patients with baseline liver metastases than in those without, confirming the similar observations that have been reported with pembrolizumab treatment, although the latter were obtained in a patient population that differed substantially from that of the current report with respect to a number of key clinical characteristics.^14^

With ORRs at 6.5 years that were stable compared with those reported at 5 years,^7^ objective responses also remained durable: median duration of response had yet to be reached at 77 months' minimum follow-up with either nivolumab-containing regimen. Indeed, in the combination group, patients with objective responses within the first 12 months of treatment had sustained PFS and OS, with only three instances of progression noted with either nivolumab-containing regimen after the 5-year analysis.

In this long-term follow-up, the overall safety profile of the three treatment regimens remained unchanged since previous reports.^4-7^ Higher incidences of AEs of all types were reported with the combination regimen, but no new safety signals or treatment-related deaths were observed.

In summary, these 6.5-year data obtained with the combination of first-line nivolumab plus ipilimumab in patients with advanced melanoma in CheckMate 067 include the longest median OS reported to date in a phase III melanoma study, as well as a median MSS that had not been reached at 77 months.

## Data Availability

Bristol Myers Squibb's policy on data sharing may be found at https://www.bms.com/researchers-and-partners/independent-research/data-sharing-request-process.html.

## References

[1] Curti BD Faries MB: Recent advances in the treatment of melanoma. N Engl J Med 384:2229-2240, 2021 34107182 10.1056/NEJMra2034861

[2] Postow MA Chesney J Pavlick AC : Nivolumab and ipilimumab versus ipilimumab in untreated melanoma. N Engl J Med 372:2006-2017, 2015 25891304 10.1056/NEJMoa1414428 PMC5744258

[3] Hodi FS Chesney J Pavlick AC : Combined nivolumab and ipilimumab versus ipilimumab alone in patients with advanced melanoma: 2-year overall survival outcomes in a multicentre, randomised, controlled, phase 2 trial. Lancet Oncol 17:1558-1568, 2016 27622997 10.1016/S1470-2045(16)30366-7 PMC5630525

[4] Larkin J Chiarion-Sileni V Gonzalez R : Combined nivolumab and ipilimumab or monotherapy in untreated melanoma. N Engl J Med 373:23-34, 2015 26027431 10.1056/NEJMoa1504030 PMC5698905

[5] Wolchok JD Chiarion-Sileni V Gonzalez R : Overall survival with combined nivolumab and ipilimumab in advanced melanoma. N Engl J Med 377:1345-1356, 2017 28889792 10.1056/NEJMoa1709684 PMC5706778

[6] Hodi FS Chiarion-Sileni V Gonzalez R : Nivolumab plus ipilimumab or nivolumab alone versus ipilimumab alone in advanced melanoma (CheckMate 067): 4-year outcomes of a multicentre, randomised, phase 3 trial. Lancet Oncol 19:1480-1492, 2018 30361170 10.1016/S1470-2045(18)30700-9

[7] Larkin J Chiarion-Sileni V Gonzalez R : Five-year survival with combined nivolumab and ipilimumab in advanced melanoma. N Engl J Med 381:1535-1546, 2019 31562797 10.1056/NEJMoa1910836

[8] Giobbie-Hurder A Gelber RD Regan MM: Challenges of guarantee-time bias. J Clin Oncol 31:2963-2969, 2013 23835712 10.1200/JCO.2013.49.5283 PMC3732313

[9] Garbe C Eigentler TK Keilholz U : Systematic review of medical treatment in melanoma: Current status and future prospects. Oncologist 16:5-24, 2011 21212434 10.1634/theoncologist.2010-0190 PMC3228046

[10] Robert C Ribas A Schachter J : Pembrolizumab versus ipilimumab in advanced melanoma (KEYNOTE-006): Post-hoc 5-year results from an open-label, multicentre, randomised, controlled, phase 3 study. Lancet Oncol 20:1239-1251, 2019 31345627 10.1016/S1470-2045(19)30388-2

[11] Robert C Grob JJ Stroyakovskiy D : Five-year outcomes with dabrafenib plus trametinib in metastatic melanoma. N Engl J Med 381:626-636, 2019 31166680 10.1056/NEJMoa1904059

[12] McArthur GA Dréno B Larkin J : 5-Year Survival Update of Cobimetinib Plus Vemurafenib in BRAF V600 Mutation–Positive Advanced Melanoma: Final Analysis of the coBRIM Study. Presented at Society for Melanoma Research (SMR), Salt Lake City, UT, November 20-23, 2019

[13] Dummer R Flaherty K Robert C : Five-year overall survival (OS) in COLUMBUS: A randomized phase 3 trial of encorafenib plus binimetinib versus vemurafenib or encorafenib in patients (pts) with *BRAF* V600-mutant melanoma. J Clin Oncol 39, 2021 (suppl 15; abstr 9507) 10.1200/JCO.21.02659 PMC9916040 35862871

[14] Tumeh PC Hellmann MD Hamid O : Liver metastasis and treatment outcome with anti–PD-1 monoclonal antibody in patients with melanoma and NSCLC. Cancer Immunol Res 5:417-424, 2017 28411193 10.1158/2326-6066.CIR-16-0325 PMC5749922

